# O.1.2-1 ACTIVE-AGE@home: a home-based functional exercise program for community dwelling frail older adults: mixed method study

**DOI:** 10.1093/eurpub/ckad133.088

**Published:** 2023-09-11

**Authors:** Dimitri Vrancken, Jade Tambeur, Emma De Keyser, Elke De Smedt, Marianne Belpaire, Sara De Cauwer, Kathleen Willems, Ine De Clerck, David Beckwée, Wim Peersman, Dominique Van de Velde, Siddhartha Lieten, Lieven Annemans, Patricia De Vriendt

**Affiliations:** Vrije Universiteit Brussel, Belgium; Artevelde University of Applied Sciences, Ghent; Vrije Universiteit Brussel, Belgium; University of Antwerp, Belgium; Vrije Universiteit Brussel, Belgium; Artevelde University of Applied Sciences, Ghent; Ghent University, Belgium; Artevelde University of Applied Sciences, Ghent; Artevelde University of Applied Sciences, Ghent; Artevelde University of Applied Sciences, Ghent; University of Antwerp, Belgium; Odisee University College, Brussels; dimitri.vrancken@arteveldehs.be; Artevelde University of Applied Sciences, Ghent; Ghent University, Belgium; Vrije Universiteit Brussel, Belgium; Vrije Universiteit Brussel, Belgium; Vrije Universiteit Brussel, Belgium; Artevelde University of Applied Sciences, Ghent; Ghent University, Belgium

## Abstract

**Purpose:**

The world population is ageing rapidly because of longer life expectancy and shrinking fertility rates. Not all older adults age in a healthy way. Evidence-based estimates indicate that 35-40% of the older adults are in a frail or prefrail state. Multi-component physical activity (PA) programs have shown to be effective to tackle frailty. However, only a scarce amount of the older adults succeed to overcome barriers towards PA. This pilot study aimed to examine the feasibility and acceptability of a home-based, functional PA program, ACTIVE-AGE@home, in frail community dwelling older adults.

**Methods:**

In a mixed method design, a single-blind pragmatic randomized trial with two intervention groups (light (n = 29) and intensive (n = 18) version of ACTIVE-AGE@home) and one control group (n = 23) was conducted. In addition, qualitative data was collected through in-depth interviews, pre-and post-intervention, to reveal the participant’s subjective experience regarding the program.

**Results:**

The arm curl test (p = 0.03), balance and gate (p = 0.052) and also independence, participation and health related quality of life (borderline significant) showed positive effects in favor for the most intensive version of ACTIVE-AGE@home compared to the control group. Nearly all participants in both intervention groups experienced positive effects regarding: fitness (90%), stability (80%), agility (70%), confidence (63%), resilient (90%) and 63% had less fear of falling. The training program was considered acceptable (95%).

**Conclusion:**

Literature showed clear impact of PA on the physical, mental, emotional and social wellbeing of older adults. These findings were confirmed in this pilot study. ACTIVE-AGE@home shows great potential for further research. Therefore, starting 2023, the effectiveness and cost-effectiveness of the updated ACTIVE-AGE@home program will be tested on a bigger scale.

**Support/funding source:**

The pilot study was developed by funding of the Flemish Government at Artevelde University of Applied Sciences. The updated version of ACTIVE-AGE@home (2022-2026) is funded by The Research Foundation – Flanders (FWO) as a Applied Biomedical Research with a Primary Social finality.

